# Quality Analysis of Stroke-Related Videos on Video Platforms: Cross-Sectional Study

**DOI:** 10.2196/80458

**Published:** 2025-11-03

**Authors:** Shao-Jie Nie, Shuai-Nan Ning, Qi-Chao Ding, Jie Hou, Yue-Yang Luo, Hai-Yang Wang

**Affiliations:** 1 Department of General Surgery, The 966th Hospital of PLA Joint Logistics Support Force, Dandong, China; 2 Chinese PLA Medical School, Beijing, China; 3 Medical Service Division, The 966th Hospital of PLA Joint Logistics Support Force, 19 Shijing Street, Zhenxing District, Dandong, 118000, China, 86 13917372167; 4 The Second Navy Hospital of Southern Threater Command of PLA, Sanya, China; 5 Changzheng Hospital, Naval Medical University, Shanghai, China; 6 Sichuan Provincial Center for Mental Health, Sichuan Provincial People's Hospital, School of Medicine, University of Electronic Science and Technology of China, Chengdu, China

**Keywords:** stroke, short videos, TikTok, video quality, PEMAT-A/V, quality assessment, Patient Education Materials Assessment Tool

## Abstract

**Background:**

Stroke has become a global public health problem due to its high incidence, disability, and mortality. In China, TikTok and Bilibili, as mainstream video-sharing platforms, serve as key sources of getting stroke-related information for people, yet their videos' content quality and reliability remain insufficiently evaluated.

**Objective:**

This cross-sectional study aimed to analyze the content and quality of stroke-related videos on Chinese video-sharing platforms.

**Methods:**

In March 2025, stroke-related videos were retrieved from TikTok and Bilibili using the search term “卒中” (Chinese for stroke). Eligible videos were analyzed for metadata and content indicators. Researchers assessed video quality using validated tools: the Global Quality Scale (GQS), modified DISCERN (mDISCERN), and Patient Education Materials Assessment Tool (PEMAT). Statistical analyses were performed with Python, including descriptive statistics, group comparisons (Kruskal–Wallis tests), and Spearman’s rank correlation to evaluate variable associations, with all *P* values adjusted for multiple comparisons using the Bonferroni method. A binary classification predictive model was developed using the random forest algorithm, accompanied by feature importance analysis.

**Results:**

Among the stroke-related videos from Bilibili (n=157) and TikTok (n=149), popular science education content predominated (204/306, 66.7%). Bilibili videos were primarily categorized as professional lectures (83/157, 52.9%), while most TikTok videos were popular science education (139/149, 93.3%). TikTok videos demonstrated significantly higher median likes and comments (*P*<.001) and shorter durations compared to Bilibili (*P*<.001). No significant differences were observed in median GQS (4) or mDISCERN scores (3) between platforms (*P*>.05). Videos produced by professional teams exhibited significantly higher GQS and PEMAT-A/V scores than those created by independent content creators (*P*<.05). Popular science education videos achieved higher PEMAT-A/V actionability scores than professional lectures (*P*<.001), while videos addressing treatment options scored lowest in GQS (*P*<.05). Strong positive correlations were identified among user engagement parameters (likes, shares, comments; ρ=0.81‐0.90, *P*<.001), but only weak correlations were found between engagement and quality scores (ρ<0.3). Machine learning modeling (AUC=0.58) identified video duration (importance score: 0.15) and uploader subscriber count (importance score: 0.13) as key predictors of content quality.

**Conclusions:**

The quality of stroke-related videos on TikTok and Bilibili remains suboptimal. Content uploaded by certified physicians and institutions received higher GQS/mDISCERN scores, confirming that medical authority is a key quality indicator. Our exploratory random-forest model, which used only basic metadata (duration, likes, subscriber count), achieved an area under the curve of 0.58, indicating that surface engagement metrics alone are insufficient to discriminate high- from low-quality material. Consequently, future screening algorithms should incorporate content-based features (eg, captions, medical keywords, visual cues) and creator credentials rather than relying solely on readily available interaction parameters. Multi-platform, larger-scale datasets are warranted to develop clinically useful prediction tools.

## Introduction

Stroke, an acute cerebrovascular disease, is pathophysiologically characterized by abrupt focal neurological deficits resulting from cerebral circulation dysfunction. Current classification delineates two entities: (1) ischemic stroke (60%‐70% of incident cases), primarily caused by atherosclerotic thrombosis or cardioembolic occlusion and (2) hemorrhagic stroke (30%‐40%), encompassing hypertensive parenchymal hemorrhage and aneurysmal subarachnoid hemorrhage [1]. Globally, stroke remained the second leading cause of death (11.6% of total deaths) and the third leading cause of death and disability combined (5.7% of total disability-adjusted life-years [DALYs]). According to the Global Burden of Disease Study 2019, there were 12.2 million incident stroke cases worldwide. Despite a 17% decline in age-standardized incidence rates from 1990 to 2019,, the absolute number of stroke cases demonstrated alarming growth—newly diagnosed cases surged by 70%, while the age-standardized prevalence rate among people under 70 years rose by 22% [2]. According to the Global Burden of Disease 2019, China maintained the highest stroke burden globally, reporting 3.94 million (95% uncertainty interval 3.43‐4.58) incident stroke cases in 2019. Alarmingly, the age-standardized incidence rate surged by 86% (73.2‐99.0) from 1990 to 2019, reaching 276.7 (241.3‐322.0) per 100,000 population by 2019 [3,4]. Notably, the mean age of patients with stroke and stroke-related fatalities in China clusters in the mid-60s, nearly a decade younger than their counterparts in high-income countries, where peak incidence occurs in the mid-70s [4]. Metabolic disorders and suboptimal lifestyle patterns, as key modifiable determinants, drive the escalating stroke burden; therefore, lifestyle modification coupled with targeted health education constitutes a cornerstone intervention for primary prevention [5,6]. Globally, approximately 50%‐70% of stroke cases are attributable to hypertension [1]. In 2019, hypertension contributed to 79.6 million DALYs, accounting for 55.5% of total stroke-related DALYs [2]. In China, the population-attributable risk of hypertension for stroke-related mortality demonstrated a sustained increase from 48.31% (1990) to 57.18% (2021) [5]. The proportion of patients with hypertension being well controlled is under 20% in China, markedly lower than the corresponding rates in the United Kingdom or the United States [4,6]. The suboptimal persistence with and adherence to secondary preventive therapies is associated with a 1-year recurrence rate of 13.2% in Chinese patients with stroke [5,7]. Therefore, given these epidemiological characteristics, systematic health education programs to improve public awareness of stroke are critically necessary. Through evidence-based health communication, such initiatives can effectively enhance preventive awareness, improve early symptom recognition, standardize prehospital emergency protocols, clarify different clinical interventions, and guide personalized rehabilitation strategies [6,8,9].

With the rapid development of mobile internet technology, video platforms—emerging communication media—have become a crucial channel for the public to access health information due to their visual presentation and fragmented dissemination features. As the world’s largest internet user market, China has seen its domestic short-video platforms, TikTok (Chinese Version) and Bilibili, surpass 700 million and 300 million monthly active users, respectively, collectively accounting for over 65% of the domestic video traffic market share [10,11]. Video-based educational interventions on digital platforms significantly enhance layperson recognition of stroke symptoms [12]. Implementation of evidence-based health education initiatives, such as the “1-2-0” stroke recognition protocol (a simplified detection method analogous to the FAST mnemonic), is associated with reduced prehospital emergency delay, thereby improving clinical outcomes and alleviating the burden among patients with stroke [13]. However, official health authorities have not fully utilized video platforms to promote health education initiatives [14], and nowadays, these open platforms lack stringent content review mechanisms, leading to inconsistent quality of disease education videos. Patients and their caregivers frequently access health-related videos for medical information. However, exposure to inaccurate content may lead to misunderstandings regarding disease diagnosis and treatment, potentially compromising personal health decision-making [15,16]. Existing studies have systematically evaluated the quality of video content on various diseases, including colorectal polyps, mitral regurgitation, and steatotic liver disease [17-19]. While studies have documented improvements in the quality of stroke-related videos on YouTube in 2023, they still fall short of serving as reliable educational resources for accurate stroke symptom recognition [20]. We conducted this cross-sectional investigation of stroke-related videos on TikTok and Bilibili to analyze their uploaded sources, content, and features, aiming to systematically evaluate videos from two platforms for their informational quality and educational value, with usage of validated quantitative scoring frameworks—including a modified version of the DISCERN instrument (mDISCERN), the Global Quality Scale (GQS), and the Patient Education Materials Assessment Tool for Audiovisual Materials (PEMAT-A/V) [21].

## Methods

### Ethical Considerations

Ethical approval was not required since this study exclusively analyzed publicly available data from short-form video-sharing platforms and involved no experimentation on human subjects. All potentially identifiable information was anonymized through strict confidentiality protocols, with data access restricted solely to the research team.

### Search Strategy and Data Collection

The search was performed on TikTok and Bilibili on March 12, 2025. To minimize bias introduced by personalized recommendation algorithms, we conducted searches using newly registered accounts on the web interface of each platform without applying any filters. The keyword “stroke” (卒中) was systematically searched across both platforms. We extracted metadata from all 196 videos displayed on the search page for TikTok. For Bilibili, we collected video metadata from the first six pages of search results, resulting in 212 videos.

Our database comprised 306 videos (157 from Bilibili and 149 from TikTok) meeting predefined inclusion criteria: stroke-related content presented in Chinese or English, excluding duplicates and irrelevant materials. Each video was recorded and analyzed across multiple parameters, including Title, Likes, Comments, Favorites, Shares, Days Published, Duration, Author Identification, Subscribers, Disease Knowledge Dimensions, and Content Typology. Platform-specific variations were noted: TikTok inherently restricts access to view counts and danmu comments (real-time bullet–screen interactions). Bilibili uniquely tracks viewer support through “Coins” donations alongside metrics like views and danmu frequency. The composite indicators of Likes, Comments, Favorites, and Shares were collectively operationalized as audience engagement metrics.

### Visual Classification

The classification framework was structured around three primary dimensions: disease knowledge dimensions, content typology, and author identification, which were subsequently treated as categorical features. The disease knowledge dimensions were categorized into six groups: symptom identification, disease prevention, pathogenesis and mechanisms, diagnostic methods, treatment options, and posttreatment rehabilitation. Content typology was systematically classified into four major types: popular science education, professional lectures, case reports or patient narratives, and other content types. For author categorization, uploader identities were divided into six hierarchical groups: certified medical professionals (platform-verified physicians), official entities (government departments, public hospitals, and state-affiliated media institutions), nonprofit science communicators (dedicated to science popularization), independent content creators, patients and family members, and other contributors.

### Assessment of Content and Quality of Videos

Video credibility was assessed using the mDISCERN, while information quality was evaluated through the GQS. The educational impact on public health literacy was quantified using the PEMAT-A/V. These three validated instruments were systematically applied to all eligible videos identified through the predefined inclusion criteria, ensuring standardized quality appraisal across the dataset.

To evaluate the reliability and quality of the videos, we employed the mDISCERN score—an adaptation of the DISCERN tool [22]. This instrument has been validated for assessing health-related video content on platforms like TikTok [23-26]. The mDISCERN score consists of 5 questions: (1) Are the aims of the video clear and achieved? (2) Are reliable sources of information used? (3) Is the information presented in a balanced and unbiased manner? (4) Are additional sources of information listed for patient reference? (5) Are areas of uncertainty mentioned? Each question is scored as 1 for “yes” and 0 for “no.” Higher scores indicate that the video is more reliable.

We applied the GQS to evaluate the quality of information in the videos. The GQS is widely recognized in assessing the quality of health information on online video platforms [23,25,26]. The GQS framework includes five key criteria: (1) poor quality (poor flow, most information missing, and not useful for patients), (2) generally poor quality (poor flow, some information provided but many important topics missing, and of very limited use to patients), (3) moderate quality (suboptimal flow, some important information adequately discussed while others are poorly covered, and somewhat beneficial for patients), (4) good quality (generally good flow, most of the relevant information is listed, though some topics are not covered and useful for patients, and (5) excellent quality (excellent flow and very useful information for patients). Higher scores indicate higher-quality videos.

To assess the educational effectiveness of the video materials for the public, we employed the PEMAT-A/V tool [27,28]. Specifically designed to evaluate audiovisual resources, the PEMAT-A/V comprises 17 items: 13 items measure the understandability of health information, while 4 items assess the actionability of recommendations. Responses are categorized as “agree” (1 point), “disagree” (0 points), or “N/A.” Scores are calculated using the formula “total points obtained divided by total possible points×100%,” generating overall scores and separate scores for understandability and actionability. Higher scores reflect superior clarity in comprehension and practical applicability of the content. The PEMAT actionability and understandability scores are also called PEMAT-A and PEMAT-U scores, respectively.

All authors are senior physicians with extensive clinical expertise in neurological specialties. One investigator (SNN) collected and archived all video data. Two independent raters (SNN and SJN) assessed the videos using the mDISCERN instrument, GQS, and PEMAT-A/V, with ratings finalized through iterative consensus discussions. Should discrepancies arise during the evaluation process, an arbiter (QCD) adjudicated the final decision, which was subsequently reviewed and calibrated by all authors to ensure unanimous approval of each rating.

The inter-rater reliability was rigorously assessed using the intraclass correlation coefficient (ICC), with reliability defined as poor (ICC<0.50), moderate (ICC=0.50‐0.75), good (ICC=0.75‐0.90), or excellent (ICC>0.90) [29]. The two researchers achieved good agreement across all evaluated domains: the GQS showed an ICC of 0.88 (95% CI 0.85‐0.90), the mDISCERN score yielded an ICC of 0.86 (95% CI 0.82‐0.88), while comprehensibility and actionability demonstrated particularly high consistency with ICC values of 0.94 (95% CI 0.93‐0.95) and 0.97 (95% CI 0.97‐0.98), respectively.

### Data Preprocessing

Systematic recoding was performed to consolidate classification variables with category frequencies below 10 into an aggregated “Others” group, then transformed into binary features via one-hot encoding. Continuous numerical variables such as Subscribers, Days Published, and Likes were retained in their original scales without normalization. The mDISCERN score (range 1‐5) served as the outcome variable, dichotomized into two quality classes: Class 0 (poor quality) for scores ≤3 and Class 1 (good quality) for scores >3.

### Statistical Analysis

This study employed the Shapiro-Wilk test to assess the normality of data derived from the included videos. As all data followed nonparametric distributions, descriptive analyses were conducted using the median. The Kruskal–Wallis test was utilized for multigroup comparisons, while the Mann-Whitney *U* test was applied for comparisons between two groups. Chi-square tests analyzed nominal categorical variables across platforms, with the Monte Carlo simulation used to calculate *P* values for categories with expected frequencies of less than five. A Bonferroni correction was applied to account for multiple hypothesis testing, with adjusted *P* values <.05 considered significant. Given the categorical nature of our dataset and non-normal distribution, Spearman’s rank correlation coefficient was adopted to evaluate parameter associations. Correlation strength was categorized as follows: <0.25 (weak), 0.25‐0.50 (moderate), 0.50‐0.75 (strong), and 0.75‐1.00 (very strong). A random forest classifier was implemented via RandomForestClassifier (scikit-learn). The dataset was partitioned into training (70%) and testing (30%) sets using stratified sampling to preserve class distribution. Data analysis and model construction were performed using Python (version 3.12.7).

## Results

### The General Characteristics of Videos

**Figure 1.**
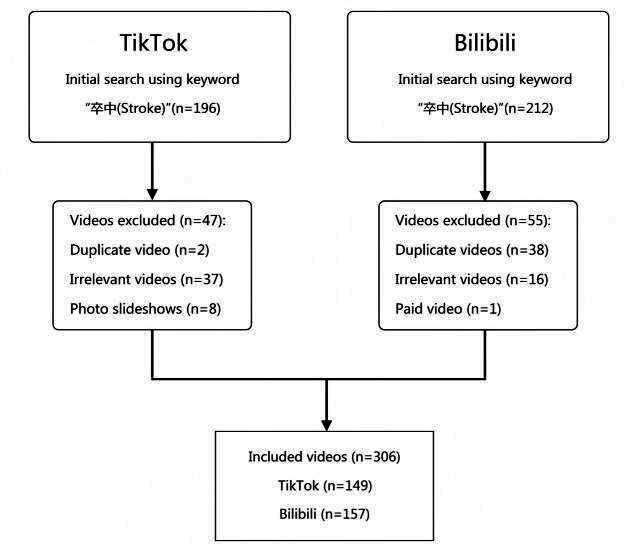
Flow diagram depicting the selection process of stroke-related videos in this analysis.

After removing duplicate and irrelevant videos, our analysis identified a final cohort of 306 eligible videos, comprising 157 from Bilibili and 149 from TikTok. Descriptive metrics demonstrated wide variability: the median video duration was 210 seconds (5‐7651 s), with median likes at 89 (0‐276,000) and median shares at 41 (0‐137,000). Comparative analyses revealed significant platform-specific disparities. Bilibili videos exhibited longer durations than their TikTok counterparts (*P*<.001). Conversely, TikTok videos achieved markedly higher median likes and comments (all *P*<.001), suggesting superior audience engagement on this platform. Temporal analysis showed TikTok videos had a median publication recency of 137 days (range 0‐1965 d), significantly shorter than Bilibili’s timeframe (*P*<.001), indicating more recent content dissemination on TikTok (Figure 1). The detailed characteristics of stroke-related videos across different platforms are summarized in Table 1.

**Table 1.** The general characteristics and scores of the stroke-related videos.

Parameters	Total (N=306)	Bilibili (n=157)	TikTok (n=149)	*P* values
Likes	89 (0‐2.76×105)	46 (0‐44,000)	210 (2‐2.76×105)	<.001
Coins	8 (0‐10,000)	8 (0‐10,000)	_^a^	_
Comments	5 (0‐8433)	2 (0‐973)	12 (0‐8433)	<.001
Favorites	86 (0‐90,000)	133 (0‐33,000)	64 (0‐90,000)	.140
Shares	41 (0‐1.37×105)	36 (0‐9553)	53 (0‐1.37×105)	.026
Views	2,627 (1‐15.67×105)	2,627 (1‐15.67×105)	_	_
Danmaku	0 (0‐1,117)	0 (0‐1,117)	_	_
Fans of video uploaders	8,930 (0‐2.6×108)	5,526 (0‐28.09×105)	3.4×104 (7‐2.6×108)	<.001
Duration (s)	210 (5‐7651)	803 (37‐7651)	84 (5‐684)	<.001
Days since upload	334 (0‐2887)	612 (0‐2887)	137 (0‐1965)	<.001
GQS^b^ score (0‐5)	4 (1-5)	4 (1-5)	4 (2-5)	.319
Modified DISCERN score (0‐5)	3 (1-5)	3 (1-5)	3 (1-5)	.443
PEMAT^c^ understandability, %	75 (18‐100)	72.73 (18‐100)	81.82 (33.33‐100)	<.001
PEMAT actionability, %	33.33 (0‐100)	33.33 (0‐100)	33.33 (0‐100)	.025

a Not available.

b GQS: Global Quality Scale.

c PEMAT: Patient Education Materials Assessment Tool.

The videos were categorized and statistically analyzed according to the classification criteria of author identification, content typology, and disease knowledge dimensions, with the results comprehensively tabulated in Table 2. Regarding author distribution, 102 videos were uploaded by certified medical professionals verified by the platforms, 109 by independent content creators, and 69 by official entities. On TikTok, medical professionals and official institutions dominated, accounting for 94.6% (141/149) of its videos, whereas independent content creators contributed 68.2% (107/157) of Bilibili videos. Regarding content typology, popular science education and professional lectures (including academic conferences, clinical courses, and guideline interpretations) constituted 66.7% (204/306) and 27.8% (85/306) of all videos, respectively. Notably, 52.9% (83/157) of Bilibili videos featured professional lectures, compared to only 1.3% (2/149) on TikTok, where popular science education dominated with 93.3% (139/149) of its content. Analysis of disease knowledge dimensions revealed that TikTok videos primarily focused on disease prevention and symptom identification (71.8% combined), while Bilibili videos predominantly addressed posttreatment rehabilitation and treatment methods (82%). Temporal and duration analyses showed that 71.8% (107/149) of TikTok videos were published after 2024, with 77.9% (116/149) lasting less than 180 seconds, whereas 60.5% (95/157) of Bilibili videos exceeded 600 seconds in duration. These findings indicate that TikTok videos are more recent and have a shorter duration than Bilibili.

**Table 2.** Description of stroke video sources, content, and characteristics.

Variable	Total (N=306), n (%)	TikTok (n=149), n (%)	Bilibili (n=157), n (%)	*P* values
Author Identification				<.001
Certified medical professionals	102 (33.3)	85 (57)	17 (10.8)	
Official entities	69 (22.5)	56 (37.6)	13 (8.3)	
Nonprofit science communicators	19 (6.2)	5 (3.4)	14 (8.9)	
Independent content creators	109 (35.6)	2 (1.3)	107 (68.2)	
Patients and family members	3 (1)	1 (0.7)	2 (1.3)	
Other contributors	4 (1.3)	0 (0)	4 (2.5)	
Content typology				<.001
Popular science education	204 (66.7)	139 (93.3)	65 (41.4)	
Professional lectures	85 (27.8)	2 (1.3)	83 (52.9)	
Case reports/patient narratives	9 (2.9)	4 (2.7)	5 (3.2)	
Other content types	8 (2.6)	4 (2.7)	4 (2.5)	
Disease knowledge dimensions				<.001
Disease prevention	78 (25.5)	54 (36.2)	24 (15.3)	
Symptom identification	64 (20.9)	53 (35.6)	11 (7)	
Posttreatment rehabilitation	56 (18.3)	10 (6.7)	46 (29.3)	
Pathogenesis and mechanisms	48 (15.7)	22 (14.8)	26 (16.6)	
Treatment options	46 (15)	10 (6.7)	36 (22.9)	
Diagnostic methods	14 (4.6)	0 (0)	14 (8.9)	
Publication date				<.001
Post-2025	70 (22.9)	53 (35.6)	17 (10.8)	
2024‐2025	98 (32)	54 (36.2)	44 (28)	
2023‐2024	63 (20.6)	25 (16.8)	38 (24.2)	
Pre-2023	75 (24.5)	17 (11.4)	58 (36.9)	
Duration				<.001
0-60 s	57 (18.6)	51 (34.2)	6 (3.8)	
60-180 s	82 (26.8)	65 (43.6)	17 (10.8)	
180-300 s	46 (15)	26 (17.4)	20 (12.7)	
300-600 s	25 (8.2)	6 (4)	19 (12.1)	
>600 s	96 (31.4)	1 (0.7)	95 (60.5)	

### Video Quality and Reliability Assessments

**Figure 2.**
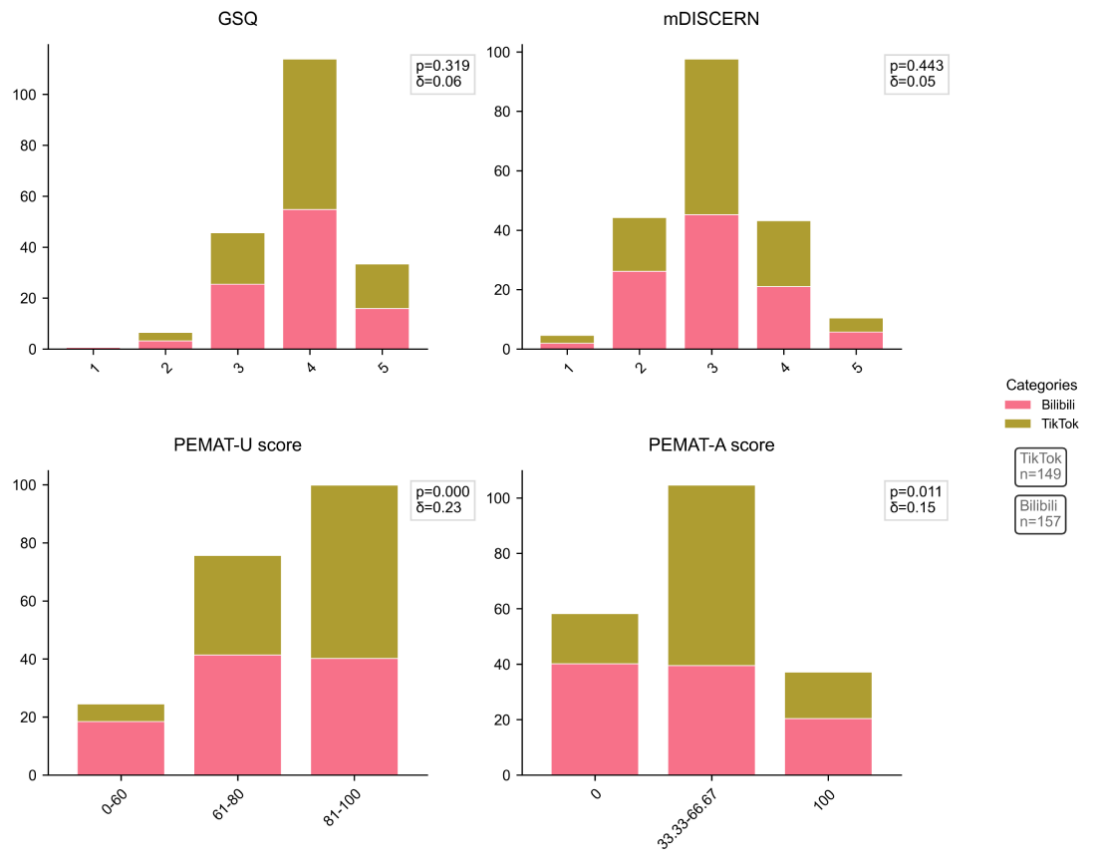
The GQS, mDISCERN scores, and PEMAT scores of the videos. GQS: Global Quality Scale; mDISCERN: modified version of the DISCERN instrument; PEMAT-A/V: Patient Education Materials Assessment Tool for Audiovisual Materials.

The videos were assessed using the mDISCERN, GQS, and PEMAT-A/V quantitative assessment tools, with detailed scoring metrics in Table 1 and Figure 2. The median scores for GQS and mDISCERN across all videos were 4 (range 1‐5) and 3 (range 1‐5), respectively, with no significant differences observed between the two platforms (*P*>.05), indicating comparable video quality across platforms but highlighting persistent limitations in content reliability. However, significant disparities were identified in PEMAT-A/V understandability (*P*<.001) and actionability (*P*=.03), demonstrating that TikTok videos were more comprehensible and actionable compared to Bilibili content. The median PEMAT-A/V actionability score across all videos was 33.33% (0%‐100%), while the median understandability score was 75% (18.18%‐100%). Despite superior understandability metrics, the markedly lower actionability scores indicate that the videos prioritized theoretical comprehension over practical implementation strategies, resulting in deficient guidance on actionable clinical steps for viewers.

### Comparison of the General Characteristics of Videos

Supplementary Table S1 and S2 in Multimedia Appendix 1 comprehensively present cross-platform intergroup comparisons of video characteristics categorized by author identification, content typology, and disease knowledge dimensions. On the TikTok platform, significant differences were observed in the publication timing of videos across various disease knowledge dimensions (*P*=.01). Videos specifically covering posttreatment rehabilitation exhibited significantly shorter publication timelines (ie, smaller video age values) compared to those focusing on pathogenesis (*P*=.04), disease prevention (*P*=.02), and symptom identification (*P*=.004). For stroke-related videos on Bilibili, videos about disease prevention were significantly shorter than those covering treatment options and diagnostic methods (*P*=.003 and *P*=.01, respectively). Additionally, significant differences were observed in shares and video duration across content typologies on Bilibili (all *P*<.001). Popular science education videos received significantly more likes and shares than professional lectures (*P*=.01 and *P*<.001, respectively), while professional lectures were significantly longer in duration (*P*<.001).

Regarding author identity on Bilibili, videos from nonprofit science communicators had significantly higher subscriber counts than those from independent content creators (*P*=.02), and videos from independent content creators were significantly longer than those from official entities (*P*=.04). On TikTok, significant differences were found in subscriber counts, likes, favorites, shares, and video duration across creators (*P*<.03). Nonprofit science communicators had significantly higher subscriber counts than certified medical professionals (*P*=.03). Furthermore, videos from nonprofit science communicators received significantly more likes, comments, favorites, and shares than those from certified medical professionals and official entities (all *P*<.006). Notably, videos from certified medical professionals on TikTok were published significantly more recently than those from official entities (*P*=.01).

Across both platforms, videos with different publication timelines exhibited significant differences in likes, favorites, comments, and shares (all *P*≤.001). On Bilibili, video duration varied significantly by publication timeline (*P*=.02). Additionally, videos with durations between 180 and 300 seconds on Bilibili received significantly more shares than those exceeding 600 seconds (*P*=.02).

### Comparative Analysis of Video Quality Assessment Scores

As shown in Supplementary Table S1 and S2 in Multimedia Appendix 1, significant differences in PEMAT actionability scores were observed across disease knowledge dimensions on both platforms (all *P*<.001). Pairwise comparisons revealed that videos addressing pathogenesis and mechanisms had significantly lower actionability scores than those covering posttreatment rehabilitation, with platform-specific significance levels (*P*=.001 for Bilibili; *P*=.004 for TikTok). On Bilibili, popular science education videos exhibited markedly higher actionability scores than professional lectures (*P*<.001).

Temporal and duration-based analyses showed significant variations in PEMAT-A/V understandability scores across video durations on both platforms (Bilibili: *P*=.0004; TikTok: *P*=.03). Specifically, Bilibili videos lasting 300‐600 seconds achieved higher actionability scores than those exceeding 600 seconds. Notably, on TikTok, videos shorter than 60 seconds demonstrated significantly higher understandability scores but lower mDISCERN reliability scores than 60‐ to 180-second videos (*P*<.01 for all comparisons), indicating a trade-off between brevity-driven accessibility and content credibility.

**Figure 3.**
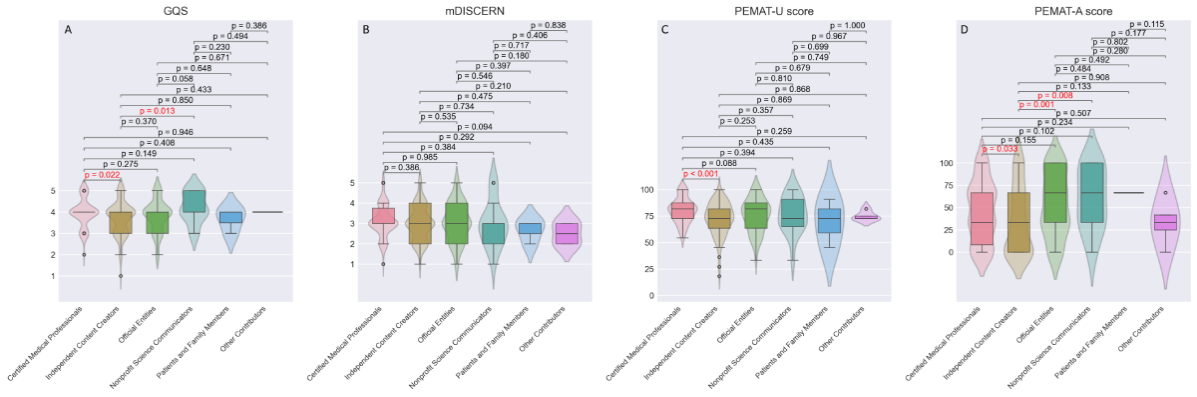
Quality assessment of videos based on author identification. (A) The GQS score. (B) The mDISCERN score. (C) The PEMAT-A/V understandability score. (D) The PEMAT-A/V actionability score. GQS: Global Quality Scale; mDISCERN: modified version of the DISCERN instrument; PEMAT-A/V: Patient Education Materials Assessment Tool for Audiovisual Materials.

After integrating video data from both platforms, comprehensive statistical analyses were conducted based on author identification, content typology, and disease knowledge dimensions, with subgroup comparisons detailed in Figures3^5^.

**Figure 4.**
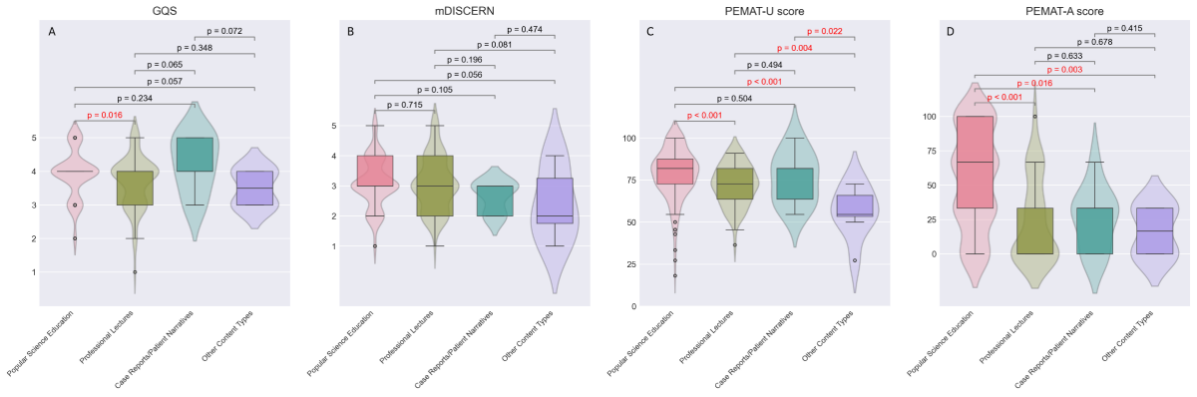
Quality assessment of videos based on content typology. (A) The GQS score. (B) The mDISCERN score. (C) The PEMAT-A/V understandability score. (D) The PEMAT-A/V actionability score. GQS: Global Quality Scale; mDISCERN: modified version of the DISCERN instrument; PEMAT-A/V: Patient Education Materials Assessment Tool for Audiovisual Materials.

**Figure 5.**
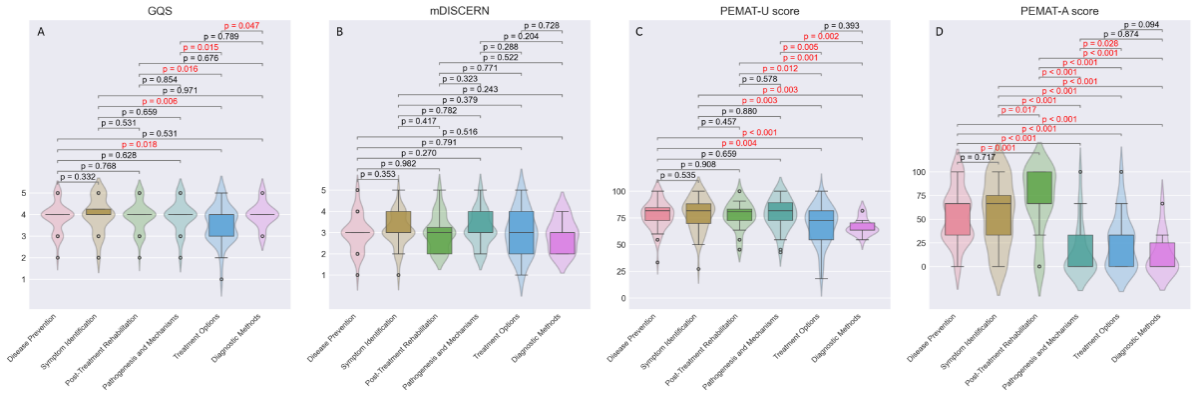
Quality assessment of videos based on disease knowledge. (A) The GQS score. (B) The mDISCERN score. (C) The PEMAT-A/V understandability score. (D) The PEMAT-A/V actionability score. GQS: Global Quality Scale; mDISCERN: modified version of the DISCERN instrument; PEMAT-A/V: Patient Education Materials Assessment Tool for Audiovisual Materials.

Certified medical professionals and nonprofit science communicators demonstrated significantly higher GQS scores than independent content creators (medical professionals versus independent creators: *P*=.02; nonprofit science communicators versus independent creators: *P*=.01). Videos produced by certified medical professionals also exhibited superior PEMAT understandability and actionability scores relative to independent content creators (*P*<.001 and *P*=.03, respectively). Similarly, videos from official entities and nonprofit science communicators achieved significantly higher PEMAT actionability scores compared to those by independent content creators (official entities versus independent creators: *P*=.001; nonprofit science communicators versus independent creators: *P*=.01). This suggests that content produced by professional teams or individuals with organizational backing provides more actionable guidance for audiences than videos created by independent contributors.

Regarding disease knowledge dimensions, videos addressing treatment methods exhibited significantly lower GQS scores than other disease-related content (all *P*<.05). Posttreatment rehabilitation videos achieved higher PEMAT actionability scores than other categories (all *P*<.05). Treatment options and diagnostic methods videos consistently scored lower in PEMAT understandability (all *P*<.05) than other disease knowledge domains.

From the perspective of content typology, popular science education videos significantly outperformed professional lectures in both GQS and PEMAT-A/V scores (all *P*<.02), particularly excelling in actionability metrics. Notably, no significant differences were observed in mDISCERN scores across subgroups.

### Correlation Analysis

**Figure 6.**
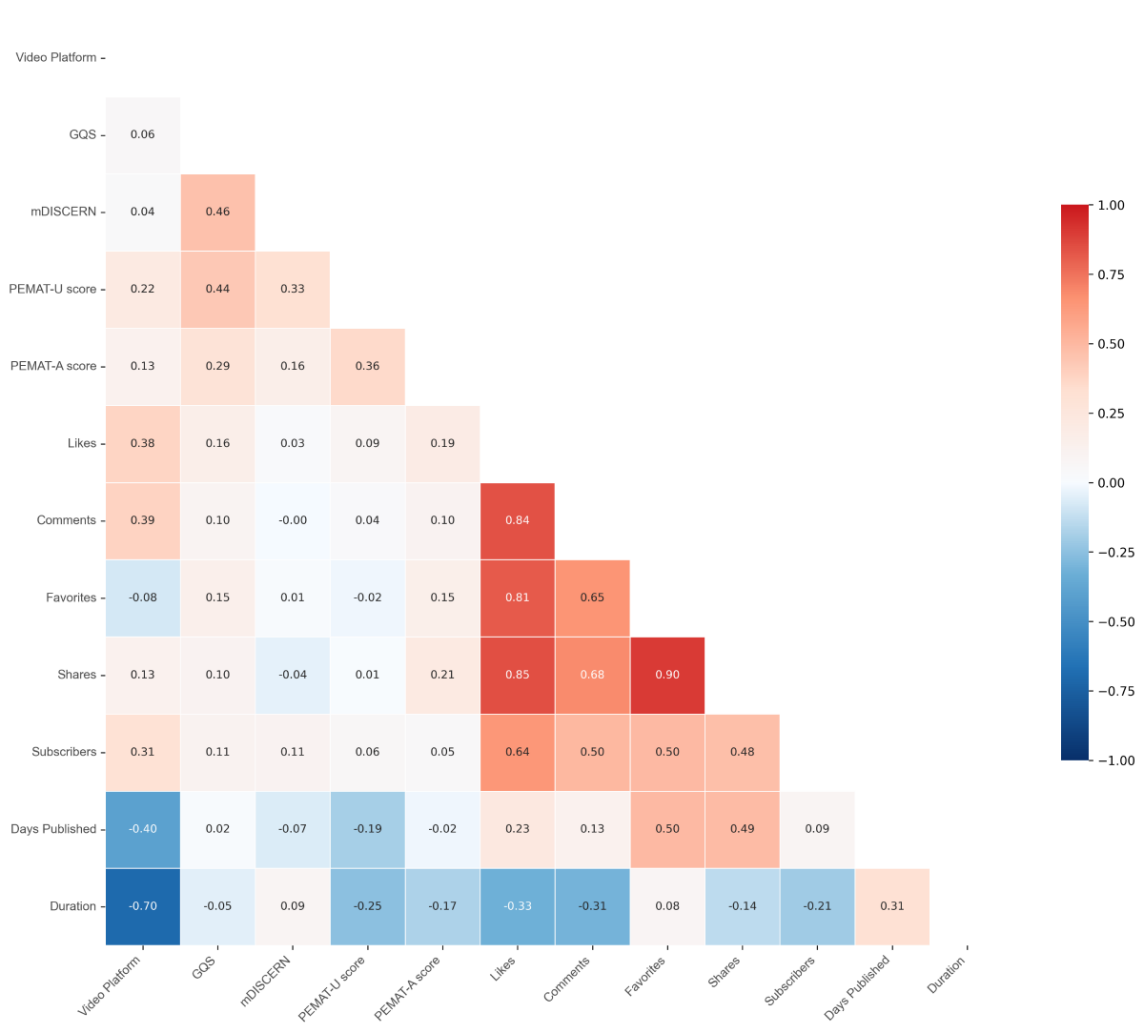
Spearman correlation between video features. GQS: Global Quality Scale; mDISCERN: modified version of the DISCERN instrument; PEMAT-A/V: Patient Education Materials Assessment Tool for Audiovisual Materials.

Spearman correlation analysis revealed the relationship between video engagement metrics and quality indicators (Figure 6). Strong positive correlations were observed between likes, shares, comments, favorites, and uploader subscriber counts (all *P*<.001), with particularly robust correlations between favorites and shares (ρ=0.90, *P*<.001), likes and shares (ρ=0.85, *P*<.001), likes and comments (ρ=0.84, *P*<.001), and likes and favorites (ρ=0.81, *P*<.001). In contrast, weaker correlations (ρ<0.3) were found between these engagement metrics and quality scores, such as GQS versus likes (ρ=0.16, *P*<.01), PEMAT-A versus likes (ρ=0.19, *P*=.001), and PEMAT-A versus shares (ρ=0.22, *P*<.001).

A moderate positive correlation was identified between video duration and publication date (ρ=0.31, *P*<.001), suggesting shorter video durations in more recent uploads. Significant negative correlations primarily involved video duration, including platform (TikTok/Bilibili) versus duration (ρ=−0.70, *P*<.001), likes versus duration (ρ=−0.33, *P*<.001), comments versus duration (ρ=−0.31, *P*<.001), PEMAT-U vs duration (ρ=−0.25, *P*<.001), and PEMAT-A versus duration (ρ=−0.17, *P*<0.001).

### Machine Learning Model

**Figure 7.**
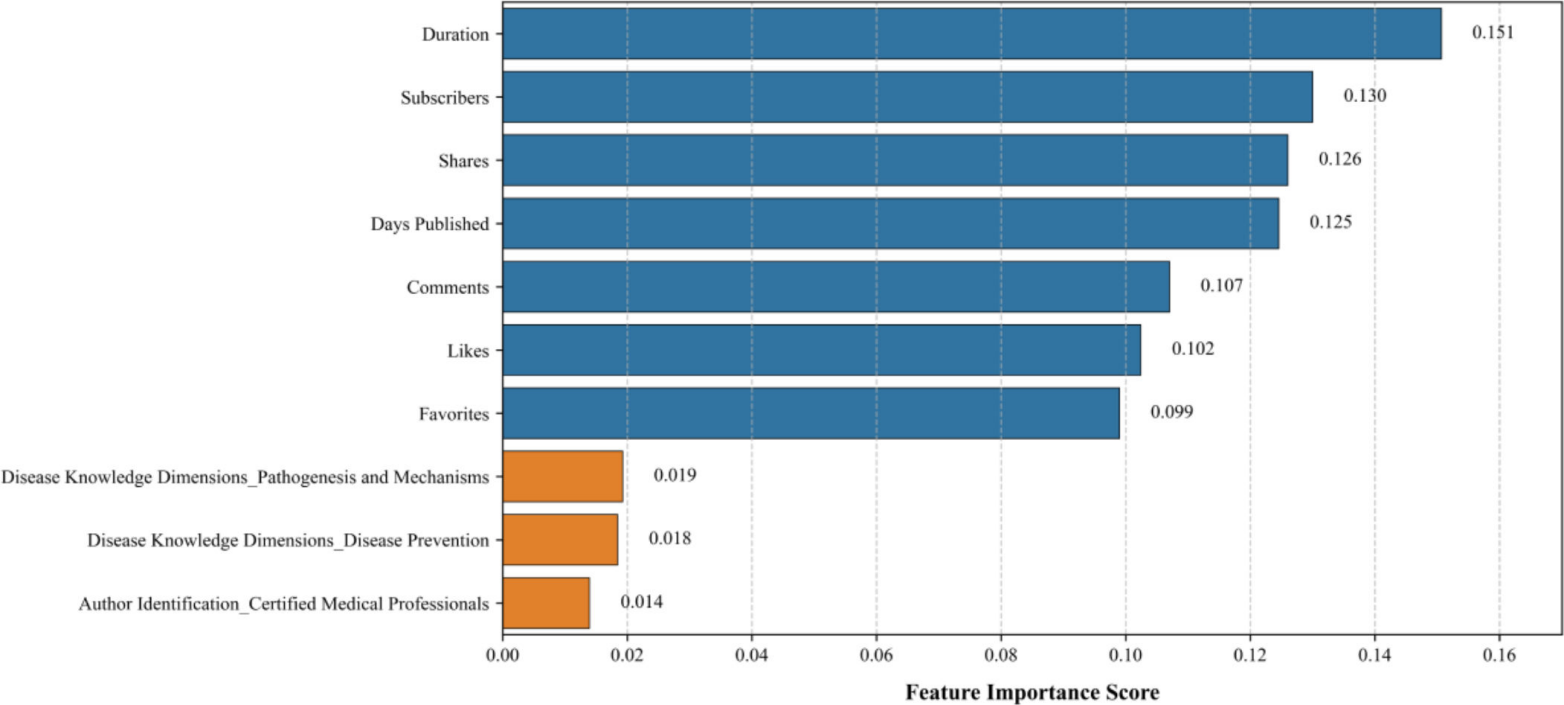
Top 10 feature importance ranking for video quality prediction model.

The model demonstrated moderate discriminative capacity, with an accuracy of 0.663 and an area under the curve (AUC)-receiver operating characteristic of 0.577, indicating limited yet nonnegligible predictive utility for video quality classification (high vs low quality). Feature importance analysis derived from the random forest model identified the top 10 predictors associated with the outcome variable (mDISCERN), with video duration (importance score: 0.151) and uploader subscribers (importance score: 0.130) emerging as the most influential factors (Figure 7).

## Discussion

### Principal Findings

Social media platforms (YouTube, TikTok, and Bilibili) have become the main channel through which the public obtains health information; yet the quality of medically themed videos is generally low, especially in specialized fields such as stroke, brain tumors, and breast cancer [30-34]. Previous studies about stroke have taken a more focused, almost exclusively on, English-language YouTube content [35], and comparative quality data for Chinese short-video platforms (TikTok and Bilibili) are lacking, although their combined user base exceeds one billion. We therefore conducted the present study.

This cross-sectional study of stroke-related videos on Bilibili and TikTok revealed several critical findings. Most videos adopted a popular science education format, which was mainly created by certified medical professionals on TikTok and independent content creators on Bilibili. Notably, many Bilibili-based independent content creators were medical students and not platform-certified physicians. Quality assessments demonstrated relatively satisfactory GQS scores, whereas mDISCERN scores remained suboptimal, indicating gaps in content reliability. Evaluation of educational utility via PEMAT metrics revealed high understandability scores but low actionability benchmarks, suggesting that while these videos are broadly comprehensible to audiences, their practical applicability remains limited. Popular science education videos demonstrated superior audience comprehension and acceptance; those targeting posttreatment rehabilitation were more likely to provide actionable procedural guidance.

### Characterization of Video Attributes and Viewer Engagement Patterns

Our study found that among the 306 analyzed videos, the highest engagement metrics reached 276,000 likes, 137,000 shares, and 90,000 favorites. This high level of audience interaction underscores the substantial public interest in stroke-related content. Additionally, the annual increase in stroke videos since 2022 reflects the growing recognition of stroke as a life-threatening condition, accompanied by gradual improvements in video quality [21].

Compared to Bilibili, TikTok videos generally achieved higher engagement metrics (likes, favorites, comments, and shares). While TikTok content predominantly originated from certified medical professionals and official entities, Bilibili videos were primarily authored by independent creators, indicating diverse stakeholder participation in stroke knowledge dissemination.

Studies have shown that short-form health education videos integrating infographics and plain-language explanations demonstrate higher audience engagement [36]. Content analysis revealed that popular science education videos dominated TikTok, whereas professional lecture videos (eg, academic conferences, guideline interpretations) prevailed on Bilibili. Notably, Bilibili videos were significantly longer than TikTok videos, aligning with its role as a long-form platform for specialized content. Thematic coverage spanned disease prevention, symptom identification, posttreatment rehabilitation, pathogenesis and mechanisms, and treatment options. This diversity fulfills the informational needs of the population at different disease stages, particularly enhancing pre-onset prevention awareness [13].

Furthermore, intergroup analyses based on classification criteria revealed significant differences in video characteristics across categories. Notably, posttreatment rehabilitation videos on TikTok had shorter days published than other disease knowledge categories, indicating a prioritization of post-stroke rehabilitation in current public health initiatives.

Science communicators demonstrated significantly higher engagement metrics (likes, favorites, comments, and shares) than certified medical professionals and official entities, though their content quality showed no statistical superiority. This discrepancy may be attributed to their larger subscriber base. A representative example includes a viral science communication video achieving 276,000 likes, 90,000 favorites, and 137,000 shares. Its success likely stems from an empathic narrative approach that contextualizes stroke risks and prevention strategies within daily life scenarios [37].

### Video Ratings and Quality Evaluation

This study utilized three validated assessment tools to evaluate the quality of all videos. Although most videos were published by certified medical professionals and official entities (eg, hospitals), the content’s overall reliability, understandability, and actionability remained suboptimal. The GQS was employed to assess comprehensive quality, yielding a median score of 4 (range 1‐5), which is higher than prior GQS evaluations of stroke-related videos [21].

The mDISCERN tool revealed moderate reliability scores (median 3), indicating acceptable but unsatisfactory credibility. Compared to previous studies analyzing videos associated with urinary tract infections, inflammatory bowel disease, and nonalcoholic fatty liver disease, stroke-related content demonstrated relatively better performance in these metrics [23-26]. Notably, TikTok videos shorter than 60 seconds exhibited significantly lower mDISCERN scores. Content analysis identified a critical gap: only 24.8% of videos cited additional information sources, resulting in zero scores for the mDISCERN criterion “Are additional sources listed for patient reference?” Adding authoritative references could enhance content credibility.

Furthermore, science communicators frequently disclosed information sources in video credits, possibly partially explaining their higher sharing rates. For instance, one science communication video achieved 137,000 shares alongside explicit citations of clinical guidelines.

The PEMAT-A/V was utilized to evaluate the educational value of stroke-related videos. The median PEMAT understandability score was 75%, while the actionability score was 33.3%. TikTok videos demonstrated significantly higher PEMAT understandability scores compared to Bilibili. Although a PEMAT score (PEMAT-U or/and PEMAT-A) above 70% indicates the content is generally reckoned to be comprehensible and actionable [27], both platforms exhibited critical deficiencies in actionability—a finding consistent with prior studies on mitral valve regurgitation-related videos [18].

Subgroup analyses revealed that videos from certified physicians achieved higher PEMAT actionability and understandability scores than those by independent content creators. Notably, videos focusing on posttreatment rehabilitation outperformed other disease knowledge categories in actionability. Additionally, popular science education videos scored significantly higher in GQS and PEMAT-A/V metrics than professional lectures.

Furthermore, some uploaders may incorporate inaccurate or misleading information to attract views. However, disseminating erroneous health content in short videos increases viewers’ risk of making health decisions based on unverified claims [15]. Therefore, audiences should exercise caution when accessing health information online and prioritize content from verified medical sources.

### Correlation Analysis and Model Prediction

Spearman’s rank correlation analysis investigated the relationships between video characteristics and quality metrics. This method enabled a comprehensive assessment of how video quality correlates with fundamental features such as engagement and duration. Strong positive correlations were observed among likes, comments, favorites, shares, and uploader subscriber counts. Conversely, significant negative correlations existed between video duration and metrics, including likes, comments, and PEMAT understandability scores. While prior studies suggest that audiences gravitate toward authoritative health content with sustained engagement, our findings revealed no robust correlations between quality scores (GQS, mDISCERN, and PEMAT) and video features (eg, duration, engagement metrics) [37]. For instance, some high-quality videos exhibited below-median engagement, potentially attributable to uploaders’ limited subscriber bases. Existing literature further implicates presentation style (eg, narrative framing) and emotional resonance as drivers of engagement, independent of content quality [37,38]. Machine learning models identified the top seven predictive features (all continuous variables: duration, likes, shares, etc), accounting for 83.7% cumulative importance in quality classification. Content attributes contributed minimally. Although baseline metrics partially reflect quality trends, neither feature-based nor content-driven models achieved clinically meaningful discriminative power. These results underscore the limitations of relying solely on engagement metrics or content taxonomies to evaluate stroke video quality.

### Recommendations Based on Our Results

Undoubtedly, health-related videos often face greater challenges in maintaining audience engagement than entertainment content due to the cognitive demands of their specialized content. Previous studies have demonstrated compelling titles paired with accurate, high-quality content can significantly enhance viewer retention [37,39]. To amplify the impact of stroke-related videos, a viable strategy involves leveraging certified medical professionals and science communication teams with substantial followings to disseminate validated disease information. Platforms should prioritize distributing content from these verified sources to broader audiences, improving public stroke awareness.

Implementing standardized content review protocols and collaboration with neurology and rehabilitation specialists could further elevate the quality and reliability of health information [40]. Such measures would establish a trustworthy repository of actionable knowledge, strengthening public understanding of stroke prevention and management. Technologically, platforms could adopt AI-powered moderation systems driven by medical knowledge graphs, akin to the Dutch model, which achieved 89% accuracy in detecting misinformation during pilot testing [41].

Future research should focus on developing interdisciplinary evaluation frameworks that integrate the GQS with information diffusion dynamics models, enabling multidimensional assessments of both medical accuracy and societal impact [21,42]. Additionally, enhancing creator authentication mechanisms would bolster audience trust in professional content.

To bridge the pervasive actionability gap (PEMAT-A median=33 %), we propose a creator-side, platform-supported intervention bundle that can be implemented immediately without altering the existing subgroup taxonomy: (i) 30-second rule: open the video with a demonstrable action (eg, FAST test) that viewers can perform in real time; (ii) Check-box animation: end with an interactive tick-list of key treatment steps; viewers tap to complete, providing creators with instant engagement analytics; (iii) Time-critical QR code: prominently display a QR code linking to a county-level map of stroke or chest-pain centers (an application programming interface can be obtained by submitting a data-use request to the National Health Commission), ensuring that audiences know where to go within the golden time window; (iv) Certified medical template: platforms should offer an optional “Medical Science” upload wizard preloaded with (a) a simplified diagnostic and treatment flowchart validated by professional societies and (b) an embedded hospital map, reducing the risk of error by nonmedical creators; (v) Social shareable: automatically generate a “learning check” completion card that the audience can post to social media, amplifying reach and reinforcing behavioral memory. Platforms should strengthen their oversight of medical science videos: they could, for example, give greater traffic weight to content that achieves ≥60% PEMAT-A, steering viewers toward videos that are both popular and behavior-changing.

Optimal video duration should match its content complexity: excessively brief videos risk oversimplification, while lengthy ones may hinder comprehension. Beyond posttreatment rehabilitation, creators should incorporate tangible, step-by-step guidance to address pervasive actionability gaps observed across other stroke knowledge domains.

### Practical Significance

To cope with stroke, one of the leading causes of mortality, we need to enhance public awareness of prevention and early recognition. In the digital era, individuals increasingly rely on video platforms to access health information and share experiences. TikTok and Bilibili, as dominant video-sharing platforms in China, have immense potential in disseminating health-related knowledge. This study represents the first comprehensive evaluation of stroke-related video content quality and reliability using multiple validated tools (GQS, mDISCERN, and PEMAT-A/V).

Our findings underscore that videos authored by certified medical professionals and official entities provide scientifically accurate and accessible content, which may improve public understanding of stroke prevention and disease management. However, persisting challenges remain, including insufficient actionable guidance and inadequate citation of credible sources, which collectively limit the practical utility of these materials. To further advance stroke literacy, platforms should prioritize distributing high-quality content while fostering collaborations among health care institutions, certified clinicians, and science communication teams to optimize content creation and dissemination strategies.

### Limitations

This study has several limitations. First, the video search and data collection were exclusively conducted on Chinese-language video-sharing platforms (TikTok and Bilibili), which may limit the generalizability of findings to other platforms or cultural contexts. Second, the analysis included only top-ranked search results for the target keyword rather than the entire corpus of available content. Furthermore, TikTok’s algorithm-driven content curation mechanisms introduce inherent variability in search result rankings, potentially compromising the representativeness of the sampled videos. Third, the cross-sectional design restricted the scope to a specific time window, failing to capture the temporal dynamics of content evolution and shifting engagement patterns on social media platforms. This temporal constraint may affect the long-term applicability and robustness of the conclusions. Future longitudinal studies are warranted to address these limitations. Fourth, the random-forest model achieved an AUC of only 0.577, indicating that readily available metadata (duration, likes, subscriber count, etc) are insufficient to predict video quality. This suboptimal discriminative performance, however, does not undermine our primary findings, which were derived from validated assessment tools (GQS, mDISCERN, and PEMAT-A/V) rather than from algorithmic prediction. The poor AUC actually reinforces our central message that engagement metrics should not serve as proxies for medical reliability. Future work will incorporate

Natural Language Processing–derived content features and creator-authority indicators to develop more accurate hybrid models. Additionally, as data were collected exclusively from Chinese-language videos on TikTok and Bilibili, our findings may not generalize to other linguistic or cultural contexts where platform algorithms, health-literacy levels, and clinical pathways differ substantially.

### Conclusions

Stroke-related videos on TikTok and Bilibili exhibit variable characters, but both show unsatisfactory quality, particularly when delivering actionable guidance for audiences. Our subgroup analysis implies that certified medical professionals convey complex medical concepts more effectively than independent creators. In addition, videos about posttreatment rehabilitation have the best actionability compared to those about other knowledge dimensions. In the further correlation analysis, we find no significant correlation between basic characteristics and video quality. Our machine learning models also fail to establish accurate predictions for quality, so we suggest not relying solely on engagement metrics or content taxonomies to judge the video’s credibility. For the public, click-heat does not equate to quality; for platforms, narrowing the gap between “what audiences watch” and “what audiences can actually learn” requires strengthened oversight of medical-science videos and traffic prioritization for videos that meet ≥60% PEMAT-A actionability. Only through such coordinated efforts—platform regulation, creator accountability—can short-video ecosystems contribute meaningfully to stroke popularization of science.

## Supplementary material

10.2196/80458 Multimedia Appendix 1 Video characteristics and quality assessment on TikTok and Bilibili across different categories.

## Abbreviations

AUC: area under the curve
DALY: disability-adjusted life-years
GQS: Global Quality Scale
ICC: intraclass correlation coefficient
mDISCERN: modified DISCERN
PEMAT-A/V: Patient Education Materials Assessment Tool for Audio-Visual Content.
