# Supplementary material for: Quality Analysis of Stroke-Related Videos on Video Platforms: Cross-Sectional Study
Source: JMIR Form Res. 2025 Nov 3;9:e80458. doi: 10.2196/80458 (PMC12582541; doi:10.2196/80458)
Supplement: Multimedia Appendix 1 [file formative-v9-e80458-s001.doc]

**Supplementary Table S1** Video characteristics and quality assessment on TikTok across different categories.

| Characteristic | n | Likes | Comments | Favorites | Shares | Uploader Subs | Video Age​ | Duration(seconds) | GQS score | mDISCERN score | Understandability(%) | Actionability(%) |
| --- | --- | --- | --- | --- | --- | --- | --- | --- | --- | --- | --- | --- |
| Disease Knowledge Dimensions | | | | | | | | | | | | |
| Pathogenesis and Mechanisms | 22 | 167.5 (75.0-864.5) | 8.0 (4.0-54.75) | 45.0 (10.75-166.75) | 34.0 (8.25-215.0) | 60,500.0 (6351.5-197,000.0) | 138.0 (67.75-462.0) | 60.5 (48.0-140.25) | 4.0 (4.0-4.0) | 3.0 (2.25-3.0) | 75.0 (72.73-84.74) | 0.0 (0.0-0.0) |
| Disease Prevention | 54 | 294.5 (50.0-2347.5) | 16.0 (4.25-96.25) | 90.5 (14.25-934.25) | 145.5 (14.75-1088.5) | 70,000.0 (2844.5-727,000.0) | 138.0 (40.5-454.0) | 87.0 (57.25-167.25) | 4.0 (4.0-4.0) | 3.0 (3.0-3.0) | 81.82 (72.73-85.71) | 0.67 (0.33-0.67) |
| Symptom Identification | 53 | 228.0 (43.0-958.0) | 10.0 (1.0-58.0) | 77.0 (13.0-626.0) | 56.0 (14.0-779.0) | 21,000.0 (1881.0-539,000.0) | 138.0 (66.0-566.0) | 76.0 (52.0-167.0) | 4.0 (4.0-4.0) | 3.0 (3.0-4.0) | 81.82 (72.73-90.91) | 0.67 (0.33-0.67) |
| Post-Treatment Rehabilitation | 10 | 79.0 (30.75-283.25) | 16.5 (3.75-22.25) | 6.0 (5.0-52.0) | 5.0 (2.25-66.25) | 982.5 (354.5-54,750.0) | 12.5 (4.25-36.75) | 69.0 (57.25-91.5) | 4.0 (4.0-5.0) | 3.0 (2.25-4.75) | 84.66 (75.0-96.88) | 0.67 (0.67-1.0) |
| Treatment Options | 10 | 205.5 (108.5-1463.0) | 18.5 (5.0-85.25) | 53.0 (11.5-389.0) | 55.5 (7.25-559.0) | 18,490.0 (3621.0-144,000.0) | 98.0 (23.5-540.5) | 134.0 (104.75-186.25) | 4.0 (3.0-4.0) | 3.0 (2.0-3.75) | 68.19 (54.55-81.82) | 0.33 (0.0-0.33) |
| P value |  | 0.55 | 0.88 | 0.26 | 0.15 | 0.27 | 0.0106 | 0.38 | 0.57 | 0.79 | 0.13 | <0.001 |
| Content Typology | | | | | | | | | | | | |
| Popular Science Education | 139 | 206.0 (46.5-1,140.0) | 11.0 (3.0-68.5) | 64.0 (10.5-458.5) | 53.0 (8.0-759.0) | 33,000.0 (1,982.5-267,500.0) | 123.0 (37.5-463.0) | 84.0 (52.0-165.5) | 4.0 (4.0-4.0) | 3.0 (3.0-4.0) | 81.82 (72.73-87.5) | 0.67 (0.33-0.67) |
| Case Reports/Patient Narratives | 4 | 3,383.5 (89.0-34,498.0) | 147.5 (22.5-1,390.25) | 337.5 (8.5-4,748.75) | 289.0 (9.25-5,925.25) | 387,487.5 (826.0-3,830,500.0) | 128.5 (46.25-294.25) | 66.0 (60.0-122.0) | 4.0 (3.75-4.25) | 3.0 (2.75-3.0) | 81.82 (81.82-86.36) | 0.33 (0.33-0.33) |
| Other Content Types | 4 | 1,989.5 (164.5-5085.25) | 48.0 (8.5-375.75) | 393.5 (28.75-1440.75) | 337.5 (20.0-829.5) | 304,500.0 (123,995.75-65,333,750.0) | 437.0 (125.75-805.5) | 163.5 (136.0-185.0) | 4.0 (3.5-4.0) | 1.5 (0.75-2.25) | 52.27 (37.5-59.09) | 0.33 (0.25-0.33) |
| Professional Lectures | 2 | 360.0 (330.5-389.5) | 45.5 (28.25-62.75) | 48.5 (31.25-65.75) | 55.5 (34.75-76.25) | 5,014,000.0 (2,521,000.0-7,507,000.0) | 147.0 (85.0-209.0) | 88.5 (81.75-95.25) | 4.5 (4.25-4.75) | 4.0 (3.5-4.5) | 70.91 (65.45-76.36) | 0.33 (0.17-0.5) |
| P value |  | 0.75 | 0.27 | 0.88 | 0.95 | 0.42 | 0.62 | 0.402 | 0.57 | 0.0285 | 0.0173 | 0.25 |
| Author Identification | | | | | | | | | | | | |
| Nonprofit Science Communicators | 5 | 64,000.0 (19,000.0-92,000.0) | 1214.0 (562.0-2794.0) | 7650.0 (3591.0-10,000.0) | 5304.0 (2382.0-30,000.0) | 952,000.0 (680,000.0-29,000,000.0) | 247.0 (71.0-847.0) | 387.0 (169.0-523.0) | 5.0 (4.0-5.0) | 3.0 (3.0-4.0) | 70.0 (63.64-81.82) | 0.33 (0.33-0.67) |
| Official Entities | 56 | 244.5 (43.75-723.75) | 6.5 (1.0-63.75) | 84.5 (13.0-394.5) | 167.0 (21.0-922.0) | 25,500.0 (6250.25-985,000.0) | 157.5 (84.75-505.25) | 88.5 (52.75-182.5) | 4.0 (3.0-4.0) | 3.0 (2.0-4.0) | 81.82 (63.64-87.5) | 0.5 (0.33-0.67) |
| Certified Medical Professionals | 85 | 195.0 (48.0-972.0) | 16.0 (5.0-49.0) | 47.0 (8.0-276.0) | 26.0 (4.0-348.0) | 33,000.0 (982.0-169,000.0) | 97.0 (19.0-430.0) | 75.0 (50.0-112.0) | 4.0 (4.0-4.0) | 3.0 (3.0-4.0) | 81.82 (72.73-87.5) | 0.33 (0.33-0.67) |
| Independent Content Creators | 2 | 17,504.5 (8756.75-26,252.25) | 827.5 (413.75-1241.25) | 1075.0 (537.5-1612.5) | 3721.5 (1862.25-5580.75) | 13,503.5 (6755.25-20,251.75) | 859.5 (444.75-1274.25) | 257.5 (249.25-265.75) | 3.5 (3.25-3.75) | 2.0 (2.0-2.0) | 72.73 (72.73-72.73) | 0.67 (0.5-0.83) |
| Patients and Family Members | 1 | 2739.0 | 152.0 | 506.0 | 767.0 | 111,000.0 | 66.0 | 281.0 | 4.0 | 3.0 | 90.91 | 0.67 |
| P value |  | 0.0107 | 0.0028 | 0.0059 | 0.0012 | 0.0278 | 0.0349 | 0.002 | 0.11 | 0.26 | 0.08 | 0.63 |
| Video Age (days) | | | | | | | | | | | | |
| 0-73 | 53 | 56.0 (20.0-210.0) | 6.0 (2.0-17.0) | 10.0 (2.0-48.0) | 7.0 (2.0-45.0) | 1669.0 (566.0-36,000.0) | _ | 75.0 (48.0-109.0) | 4.0 (4.0-4.0) | 3.0 (3.0-4.0) | 81.82 (75.0-87.5) | 0.33 (0.33-0.67) |
| 73-439 | 54 | 191.5 (88.75-1133.25) | 11.0 (4.0-80.75) | 60.0 (15.5-575.25) | 43.0 (14.0-559.5) | 77,000.0 (11,250.0-932,250.0) | _ | 79.0 (51.25-163.25) | 4.0 (4.0-4.0) | 3.0 (3.0-3.0) | 81.82 (70.68-87.5) | 0.33 (0.33-0.67) |
| 439-805 | 25 | 1711.0 (510.0-1,1000.0) | 48.0 (9.0-457.0) | 426.0 (172.0-2818.0) | 985.0 (348.0-1432.0) | 445,000.0 (8545.0-2,220,000.0) | _ | 95.0 (63.0-168.0) | 4.0 (4.0-4.0) | 3.0 (3.0-3.0) | 72.73 (72.73-81.82) | 0.67 (0.33-0.67) |
| ＞805 | 17 | 957.0 (312.0-15,000.0) | 37.0 (14.0-482.0) | 308.0 (160.0-832.0) | 518.0 (243.0-4455.0) | 164,000.0 (19,000.0-539,000.0) | _ | 112.0 (57.0-235.0) | 4.0 (3.0-4.0) | 3.0 (2.0-3.0) | 72.73 (63.64-81.82) | 0.33 (0.33-0.67) |
| P value |  | <0.001 | <0.001 | <0.001 | <0.001 | <0.001 | _ | 0.38 | 0.74 | 0.66 | 0.0453 | 0.23 |
| Duration(seconds) | | | | | | | | | | | | |
| 0-60 | 51 | 375.0 (74.0-1733.0) | 16.0 (4.0-78.5) | 96.0 (13.0-680.5) | 56.0 (4.5-578.5) | 99,000.0 (5107.0-509,500.0) | 113.0 (26.5-385.0) | _ | 4.0 (3.5-4.0) | 3.0 (2.0-3.0) | 87.5 (78.41-100.0) | 0.33 (0.17-0.67) |
| 60-180 | 65 | 195.0 (48.0-592.0) | 10.0 (4.0-38.0) | 50.0 (10.0-172.0) | 34.0 (11.0-393.0) | 12,000.0 (1881.0-164,000.0) | 138.0 (37.0-473.0) | _ | 4.0 (4.0-4.0) | 3.0 (3.0-4.0) | 81.82 (70.0-81.82) | 0.33 (0.33-0.67) |
| 180-300 | 26 | 127.0 (35.75-2293.75) | 14.0 (1.25-79.75) | 75.5 (10.25-721.25) | 52.5 (9.5-1332.25) | 22,000.0 (2844.5-154,500.0) | 137.5 (69.5-539.5) | _ | 4.0 (3.0-4.0) | 3.0 (3.0-3.0) | 80.91 (72.73-81.82) | 0.67 (0.33-1.0) |
| 300-600 | 6 | 2915.5 (697.25-5436.75) | 104.0 (35.25-148.75) | 1527.0 (208.5-3368.25) | 992.5 (290.25-1393.25) | 441,500.0 (65,000.0-884,000.0) | 210.0 (96.5-938.5) | _ | 4.0 (4.0-4.75) | 3.5 (3.0-4.0) | 72.73 (65.91-79.55) | 0.17 (0.0-0.33) |
| ＞600 | 1 | 19,000.0 | 562.0 | 7650.0 | 5304.0 | 54,000.0 | 38.0 | _ | 5.0 | 4.0 | 81.82 | 0.67 |
| P value |  | 0.19 | 0.25 | 0.15 | 0.23 | 0.39 | 0.43 | _ | 0.26 | 0.004 | <0.001 | 0.0332 |

**Supplementary Table S2** Video characteristics and quality assessment on Bilibili across different categories.

| Characteristic | n | Likes | Comments | Favorites | Shares | Uploader Subs | Video Age | Duration(seconds) | GQS score | mDISCERN score | Understandability(%) | Actionability(%) |
| --- | --- | --- | --- | --- | --- | --- | --- | --- | --- | --- | --- | --- |
| Disease Knowledge Dimensions | | | | | | | | | | | | |
| Symptom Identification | 11 | 13.0 (11.0-204.5) | 1.0 (0.5-2.5) | 54.0 (21.5-766.5) | 32.0 (15.5-224.5) | 8929.0 (3523.5-52000.0) | 863.0 (258.5-1108.5) | 766.0 (557.5-1371.0) | 4.0 (3.0-5.0) | 3.0 (2.0-3.5) | 63.64 (54.55-81.82) | 0.0 (0.0-1.0) |
| Disease Prevention | 24 | 42.0 (10.0-114.25) | 1.0 (0.0-4.75) | 58.0 (21.25-212.5) | 63.0 (8.0-238.75) | 674.5 (45.5-25,500.0) | 521.0 (255.0-1116.75) | 299.5 (210.75-664.25) | 4.0 (3.75-4.0) | 3.0 (2.0-3.0) | 72.73 (63.64-81.82) | 0.33 (0.25-0.67) |
| Pathogenesis and Mechanisms | 26 | 53.5 (19.25-397.75) | 5.0 (0.0-27.75) | 168.5 (25.75-523.75) | 32.5 (8.0-254.75) | 8472.5 (4870.0-67,000.0) | 827.0 (205.25-1451.5) | 893.5 (374.25-1917.5) | 4.0 (4.0-4.0) | 3.0 (3.0-4.0) | 81.82 (72.73-90.91) | 0.0 (0.0-0.33) |
| Post-Treatment Rehabilitation | 46 | 62.5 (25.25-146.75) | 3.0 (1.0-11.0) | 176.0 (57.5-454.0) | 67.0 (12.25-185.5) | 2602.5 (455.5-7104.75) | 629.5 (311.0-1027.5) | 529.5 (162.75-2174.5) | 4.0 (4.0-4.0) | 3.0 (2.25-3.0) | 72.73 (72.73-81.82) | 0.67 (0.67-1.0) |
| Treatment Options | 36 | 16.5 (8.5-106.75) | 1.0 (0.0-4.75) | 64.5 (29.0-205.5) | 17.5 (5.0-48.5) | 6329.0 (2247.0-30,250.0) | 389.0 (176.75-691.25) | 1366.5 (638.0-1981.75) | 3.5 (3.0-4.0) | 3.0 (2.0-4.0) | 72.73 (54.55-81.82) | 0.33 (0.0-0.33) |
| Diagnostic Methods | 14 | 55.5 (10.75-200.25) | 3.5 (0.25-5.75) | 234.5 (26.75-817.75) | 36.5 (2.5-128.0) | 9202.5 (4440.0-17,000.0) | 580.0 (123.75-893.25) | 1813.0 (1211.0-2654.75) | 4.0 (4.0-4.0) | 3.0 (2.0-3.0) | 63.64 (63.64-70.46) | 0.0 (0.0-0.25) |
| P value |  | 0.46 | 0.17 | 0.32 | 0.34 | 0.0149 | 0.46 | 0.0025 | 0.06 | 0.35 | 0.0139 | <0.001 |
| Content Typology | | | | | | | | | | | | |
| Professional Lectures | 83 | 19.0 (8.5-74.0) | 1.0 (0.0-5.0) | 63.0 (23.0-293.5) | 17.0 (4.0-39.5) | 6329.0 (2020.0-15,000.0) | 599.0 (161.5-812.0) | 1685.0 (1061.5-2838.0) | 4.0 (3.0-4.0) | 3.0 (2.0-4.0) | 72.73 (63.64-81.82) | 0.0 (0.0-0.33) |
| Popular Science Education | 65 | 84.0 (25.0-263.0) | 4.0 (1.0-28.0) | 174.0 (56.0-576.0) | 153.0 (28.0-294.0) | 3224.0 (127.0-56,000.0) | 727.0 (138.0-1374.0) | 294.0 (143.0-501.0) | 4.0 (4.0-4.0) | 3.0 (2.0-4.0) | 81.82 (66.67-90.91) | 0.67 (0.33-1.0) |
| Case Reports/Patient Narratives | 5 | 46.0 (12.0-1490.0) | 3.0 (1.0-39.0) | 186.0 (30.0-970.0) | 48.0 (45.0-278.0) | 18,000.0 (23.0-39,000.0) | 1032.0 (863.0-1283.0) | 523.0 (279.0-614.0) | 5.0 (4.0-5.0) | 3.0 (2.0-3.0) | 63.64 (63.64-63.64) | 0.0 (0.0-0.33) |
| Other Content Types | 4 | 163.0 (19.5-1385.5) | 1.5 (0.0-210.75) | 94.0 (12.5-643.5) | 35.5 (13.25-595.0) | 299,123.5 (1685.75-640,250.0) | 515.0 (353.0-718.0) | 425.0 (180.0-835.0) | 3.0 (3.0-3.25) | 2.0 (1.75-2.5) | 59.09 (47.73-65.91) | 0.0 (0.0-0.08) |
| P value |  | 0.0031 | 0.07 | 0.11 | <0.001 | 0.86 | 0.07 | <0.001 | 0.0061 | 0.3 | 0.0292 | <0.001 |
| Author Identification | | | | | | | | | | | | |
| Certified Medical Professionals | 17 | 247.0 (13.0-1309.0) | 9.0 (0.0-56.0) | 234.0 (23.0-1242.0) | 63.0 (4.0-292.0) | 8158.0 (1990.0-86,000.0) | 607.0 (89.0-1194.0) | 282.0 (154.0-1340.0) | 4.0 (3.0-4.0) | 3.0 (3.0-3.0) | 72.73 (63.64-81.82) | 0.33 (0.0-0.67) |
| Independent Content Creators | 107 | 38.0 (10.0-100.0) | 2.0 (0.0-6.0) | 113.0 (31.5-330.0) | 26.0 (7.0-113.5) | 4461.0 (463.0-10,748.5) | 602.0 (218.0-909.0) | 1169.0 (353.0-2260.0) | 4.0 (3.0-4.0) | 3.0 (2.0-4.0) | 72.73 (63.64-81.82) | 0.33 (0.0-0.67) |
| Official Entities | 13 | 46.0 (11.0-301.0) | 0.0 (0.0-2.0) | 26.0 (13.0-221.0) | 49.0 (15.0-294.0) | 759.0 (342.0-538,000.0) | 863.0 (137.0-1233.0) | 325.0 (276.0-517.0) | 4.0 (4.0-5.0) | 4.0 (2.0-4.0) | 72.73 (63.64-81.82) | 0.67 (0.0-1.0) |
| Nonprofit Science Communicators | 14 | 146.0 (23.75-3547.5) | 9.0 (1.0-44.25) | 272.0 (58.25-855.5) | 140.5 (32.0-277.0) | 41,000.0 (10,402.5-549,000.0) | 546.5 (245.75-1302.0) | 532.5 (320.0-683.75) | 4.0 (4.0-4.75) | 3.0 (2.0-3.0) | 77.28 (68.19-90.91) | 0.67 (0.33-1.0) |
| Official Entities | 4 | 17.5 (8.75-34.5) | 1.0 (0.75-1.5) | 94.0 (12.5-175.0) | 32.5 (7.5-58.5) | 36,623.5 (1806.75-73,250.0) | 657.0 (516.75-707.5) | 1297.0 (1067.5-2320.0) | 4.0 (4.0-4.0) | 2.5 (2.0-3.0) | 72.73 (72.73-75.0) | 0.33 (0.25-0.42) |
| Patients and Family Members | 2 | 522.5 (290.25-754.75) | 36.0 (23.5-48.5) | 1167.5 (626.75-1708.25) | 1127.0 (596.5-1657.5) | 3594.5 (3409.25-3779.75) | 1293.5 (1166.25-1420.75) | 429.5 (273.75-585.25) | 3.5 (3.25-3.75) | 2.5 (2.25-2.75) | 59.09 (52.27-65.91) | 0.67 (0.67-0.67) |
| P value |  | 0.07 | 0.0498 | 0.32 | 0.07 | 0.0422 | 0.7 | 0.0024 | 0.13 | 0.46 | 0.57 | 0.05 |
| Video Age (days) | | | | | | | | | | | | |
| 0-73 | 17 | 2.0 (0.0-13.0) | 0.0 (0.0-1.0) | 17.0 (0.0-34.0) | 2.0 (0.0-6.0) | 127.0 (115.0-8929.0) | _ | 943.0 (295.0-1957.0) | 4.0 (4.0-4.0) | 3.0 (3.0-4.0) | 72.73 (72.73-81.82) | 0.33 (0.0-0.67) |
| 73-439 | 44 | 16.0 (7.75-70.5) | 1.0 (0.0-3.25) | 45.5 (9.0-167.25) | 8.0 (2.75-36.5) | 6466.0 (969.25-27,000.0) | _ | 954.5 (338.5-1892.0) | 4.0 (3.0-4.0) | 3.0 (2.0-3.25) | 72.73 (63.64-81.82) | 0.17 (0.0-0.67) |
| 439-805 | 38 | 66.5 (20.5-162.25) | 4.0 (1.0-11.0) | 230.0 (63.5-429.5) | 49.0 (21.0-185.5) | 5927.5 (629.75-20,250.0) | _ | 1439.0 (577.25-2386.5) | 4.0 (3.0-4.0) | 3.0 (3.0-4.0) | 72.73 (63.64-87.12) | 0.67 (0.0-0.67) |
| ＞805 | 58 | 77.5 (31.5-291.5) | 4.0 (1.0-24.0) | 189.0 (86.25-784.75) | 121.5 (33.5-276.75) | 4870.0 (591.75-43,000.0) | _ | 509.0 (230.25-1199.75) | 4.0 (4.0-4.0) | 3.0 (2.0-3.0) | 72.73 (63.64-81.82) | 0.33 (0.0-0.67) |
| P value |  | <0.001 | <0.001 | <0.001 | <0.001 | 0.06 | _ | 0.0242 | 0.29 | 0.63 | 0.9 | 0.2 |
| Duration(seconds) | | | | | | | | | | | | |
| 0-60 | 6 | 72.5 (17.0-155.0) | 3.0 (0.5-10.0) | 142.5 (40.25-289.0) | 184.5 (41.0-232.75) | 330.0 (96.25-4434.5) | 400.0 (305.75-1149.0) | _ | 4.0 (4.0-4.0) | 2.5 (2.0-3.0) | 73.34 (57.58-86.67) | 0.33 (0.33-0.58) |
| 60-180 | 17 | 84.0 (25.0-225.0) | 3.0 (1.0-6.0) | 86.0 (41.0-253.0) | 63.0 (16.0-163.0) | 3224.0 (399.0-77,000.0) | 607.0 (306.0-868.0) | _ | 4.0 (4.0-4.0) | 3.0 (3.0-4.0) | 72.73 (72.73-81.82) | 0.67 (0.0-1.0) |
| 180-300 | 20 | 86.0 (16.25-489.75) | 4.0 (0.0-29.0) | 195.5 (42.0-1124.5) | 168.0 (38.0-389.5) | 2296.0 (81.5-87,750.0) | 997.0 (139.25-1517.25) | _ | 4.0 (3.0-4.0) | 3.0 (2.0-3.0) | 72.73 (63.64-81.82) | 0.67 (0.33-0.75) |
| 300-600 | 19 | 90.0 (13.5-531.5) | 3.0 (0.5-33.5) | 221.0 (20.5-948.0) | 167.0 (23.5-300.5) | 3590.0 (201.5-58,500.0) | 870.0 (423.0-1411.0) | _ | 4.0 (4.0-5.0) | 3.0 (2.0-3.5) | 72.73 (59.09-90.91) | 1.0 (0.33-1.0) |
| ＞600 | 95 | 33.0 (10.0-79.0) | 1.0 (0.0-5.5) | 92.0 (25.5-329.0) | 23.0 (5.0-67.0) | 6329.0 (1990.0-15,000.0) | 602.0 (161.5-842.0) | _ | 4.0 (3.0-4.0) | 3.0 (2.0-4.0) | 72.73 (63.64-81.82) | 0.0 (0.0-0.67) |
| P value |  | 0.11 | 0.38 | 0.6 | 0.0055 | 0.74 | 0.26 | _ | 0.28 | 0.35 | 0.85 | <0.001 |
